# Stress Distribution on Prepared Tooth With Shoulder and Radial Shoulder Margin to Receive Crowns of Three Different Materials: A Finite Element Analysis

**DOI:** 10.7759/cureus.55538

**Published:** 2024-03-05

**Authors:** Shipha Hegde, Anamika Deb, Ban A Almudarris, Rajkiran Chitumalla, Shashank Jaiswal, Satheesh R, Ramesh K Nadiger, Gouri V Anehosur

**Affiliations:** 1 Prosthodontics and Crown and Bridge, A B Shetty Memorial Institute of Dental Sciences, Mangalore, IND; 2 Oral and Maxillofacial Pathology and Microbiology and Forensic Odontology, Bhabha College of Dental Sciences, Bhopal, IND; 3 Restorative and Prosthodontic Unit, College of Dentistry, City University Ajman, Ajman, ARE; 4 Restorative and Prosthetic Dental Sciences, College of Dentistry, King Saud bin Abdulaziz University for Health Sciences, King Abdullah International Medical Research Center, Ministry of National Guard Health Affairs, Riyadh, SAU; 5 Orthodontics, Chhatrapati Shahu Maharaj Shikshan Sanstha (CSMSS) Dental College & Hospital, Aurangabad, IND; 6 Prosthodontics and Crown and Bridge, Maulana Azad Institute of Dental Sciences, New Delhi, IND; 7 Prosthodontics and Crown and Bridge, Shri Dharmasthala Manjunatheshwara (SDM) College of Dental Sciences & Hospital, Dharwad, IND

**Keywords:** finite element analysis (fea), von-mises stress, maximum principal stress, radial shoulder margin, peek

## Abstract

Aim and background

This study aims to determine the stress distribution on the prepared tooth at the margins with shoulder and radial shoulder finish lines when an occlusal load of 300N was applied to ceramic, zirconia, and polyether ether ketone (PEEK) crowns.

Materials and methods

Six models of mandibular first molar teeth were fabricated. The tooth models were subdivided into two groups with shoulder and radial shoulder margins, respectively (n = 18). The teeth were restored with three different prosthetic crown materials (ceramic, zirconia, and PEEK). To simulate the typical forces experienced by a prosthetic crown material in a lower posterior tooth during chewing and biting, an occlusal load of 300N was applied to each of the samples, and the maximum principal stress (Pmax) and von Mises stress were calculated, respectively. These samples were then compared and evaluated to determine the material best suited as a prosthetic crown material of choice for a lower posterior tooth.

Results

Among the materials used, the maximum principal stress value was the least in PEEK crowns. The von Mises stress value was highest for the zirconia crown with shoulder margin and was least for the PEEK crown with a similar margin.

Conclusion

PEEK as a crown material was found to be a better choice for lower posterior teeth as there was the least maximum principal stress at the margin, irrespective of either shoulder or radial shoulder finish line used.

## Introduction

Dental crowns that look and function like natural teeth have long been sought. The success of these crowns depends on several factors, such as the material of the crown, cementation type, marginal type, marginal adaptation, preparation design, and functional and parafunctional activities [1].

The marginal fit or accuracy of restoration is best defined in terms of “misfit,” or the gap measured at various points between the restoration and the tooth [2]. The classic shoulder is the finish line of choice for the mentioned restorative materials, with its wide ledge providing resistance to occlusal forces, thereby reducing the stress that might lead to the fracture of the restorative material. Radial shoulder, a modification of the shoulder finish line, uses a coarse flat-end tapered diamond for initial instrumentation with the fine variety of the bur for creating a small radius internal angle. A special biangle chisel is used for finishing the margin, which reduces stress compared to the classic shoulder margin [3]. Knowledge of factors that influence stress and its distribution is of key importance to the successful production of durable restorations. One of these is the marginal geometry [4].

All-ceramic and zirconia crowns are frequently employed today due to the rising demand for aesthetics and biocompatibility in restorative materials, while polyether ether ketone (PEEK) crowns are still in their early stages of clinical application. Ceramic crowns, being devoid of a metallic core or substructure, have replaced metal and metal-ceramic restorations due to their aesthetic superiority, with studies concluding that these crowns are feasible as posterior restorations when a favorable marginal finish line is provided, resulting in adequate stress distribution.

Various studies have also concluded that the finish line height and position have a great impact on the periapical stress on these crowns. Of the other ceramic systems, zirconia has mechanical properties similar to those of metal. With further advancement of materials, PEEK crowns have also begun to be used as posterior tooth restorative materials due to their mechanical properties being similar to those of dentin and enamel, providing superior marginal integrity and making them advantageous over ceramic crowns. This implies that for the long-term performance of crowns made of various materials, the crown material, finish line, and stress distribution at these finish lines are crucial.

A three-dimensional (3D) finite element analysis (FEA) study is an effective tool for analyzing and understanding the mechanical behavior of live tissue and organs against forces, making it suitable for determining the marginal integrity and stress distribution on crowns of different materials with different finish lines [1]. The FEA method has several advantages over other methods, including the fact that solid objects with complex geometry can be modeled with realistic assumptions about the material, allowing a realistic model to be created using software. Thirdly, stress distribution and displacements can be obtained numerically [1]. Hence, the accuracy of FEA is based on how correct the assumption on the models is with their ability to simulate actual physical conditions, herewith the action of mastication.

Although various individual studies have been conducted to determine the marginal integrity and stress distribution of ceramic and zirconia crowns, comparative analysis for the abovementioned criteria for ceramic, zirconia, and PEEK crowns has not been conducted. Hence, the assessment of the same and determining the appropriate metal-free restorative crown material for posterior teeth was the primary aim of this study.

## Materials and methods

This study was conducted to determine the influence of “shoulder” and “radial shoulder” finish lines on stress distribution at the margin of a prepared mandibular first molar tooth using 3D FEA in the Department of Prosthodontics, Shri Dharmasthala Manjunatheshwara (SDM) College of Dental Sciences & Hospital, Dharwad, India.

The study was done using a 3D FEA on a workstation computer with the following configuration: an Intel Core 2 Duo with 2.1 GHz, 2 GB of RAM, a 2 GB graphics card, a 320 GB hard disk, and a 17″ monitor [5].

An X-force/SH spiral CT scan machine was used for taking the CT scan images of the cranium with Materialise Interactive Medical Image Control System (MIMICS) 8.11, a medical modeling software used for the visualization and segmentation of CT/MRI images. Analysis System Software (ANSYS) 12.1 was used for carrying out the FEA of structures and fluids, which has various applications in the automotive, civil, manufacturing, aerospace, and biomedical fields. The HyperMesh 13.0 software was used for converting geometric models into finite element models, with Altair HyperWorks being an engineering framework for product design that maximizes product performance, automates design processes, and improves profitability within an open and flexible environment.

The following procedures were followed for carrying out the FEA:

The CT scan of a patient was procured and then converted to Digital Imaging and Communications in Medicine (DICOM) data and geometric models using reverse engineering techniques. Reverse engineering includes scanning the models and measuring the length, diameter, and other features using standard measuring instruments and scanning machines. In our study, the CT scan data was processed, and only the region of interest for the study, the mandible, was extracted first, and then from that again, we extracted the first molar along with the surrounding bone structure. This data on the extracted mandibular first molar was exported in Standard Tessellation Language (STL) format for further action.

The STL file was imported into HyperMesh software and converted into the geometric model (consisting of only surface data). The geometric model of the mandibular first molar with surrounding bone structure was then converted into an FE model. In HyperMesh, the individual parts like soft bone, hard bone, dentine, and crown were then discretized (meshing) and assembled. Meshed models are called finite element models, and they consist of nodes and element data.

The crown was prepared with an occlusal surface reduction between 1.5 mm and 2 mm and a 1.5 mm axial reduction, and internal line angles were rounded and tapered with a flat-ended diamond to create a good margin. Crown preparation was done on the patient tooth as per guidelines for respected crown material, following which the prepared tooth was scanned (CBCT taken) for fabrication of the crown via computer-aided design/computer-aided manufacturing. Three shoulder and three radial shoulder margin models were made in total, and they were each further analyzed with three different materials under a 300N stress [1]. The specific reason for applying an occlusal load of 300N to each of the samples is to simulate the typical forces experienced by a prosthetic crown material in a lower posterior tooth during chewing and biting. Once the model was prepared, material properties for each part were obtained, and FEA was conducted (Table 1).

**Table 1:** Material properties used

Variables	Modulus of elasticity (GPa)	Flexural strength	Poisson ratio
Ceramic (IPS Emax, Ivoclar)	95 GPa	360 MPa	0.3
Zirconia	94.5 GPa	230 MPa	0.34
PEEK	3-4 GPa	87-95.2 MPa	0.3779

The occlusal surface was loaded, and the bottom part of the bone was fastened. We then exported the FE model with loads and boundary conditions to carry out the linear static analysis using ANSYS software. Post-processing the results and documentation was the last step in this study.

## Results

The data obtained from FE analysis is shown in stress maps with a color scale, making it possible to compare the stress distribution in different structures (crown and margins of preparation) of the 18 models when occlusal forces of 300N were applied to a tooth having crowns of ceramic, zirconia, and PEEK with radial shoulder and shoulder margin.

**Figure 1:**
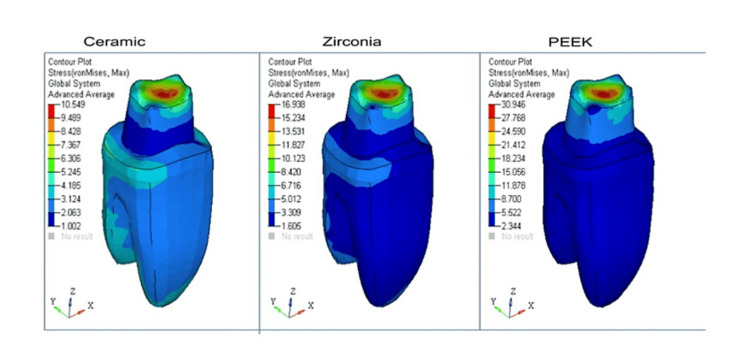
Radial shoulder marginal von Mises stress contours (MPa)

**Figure 2:**
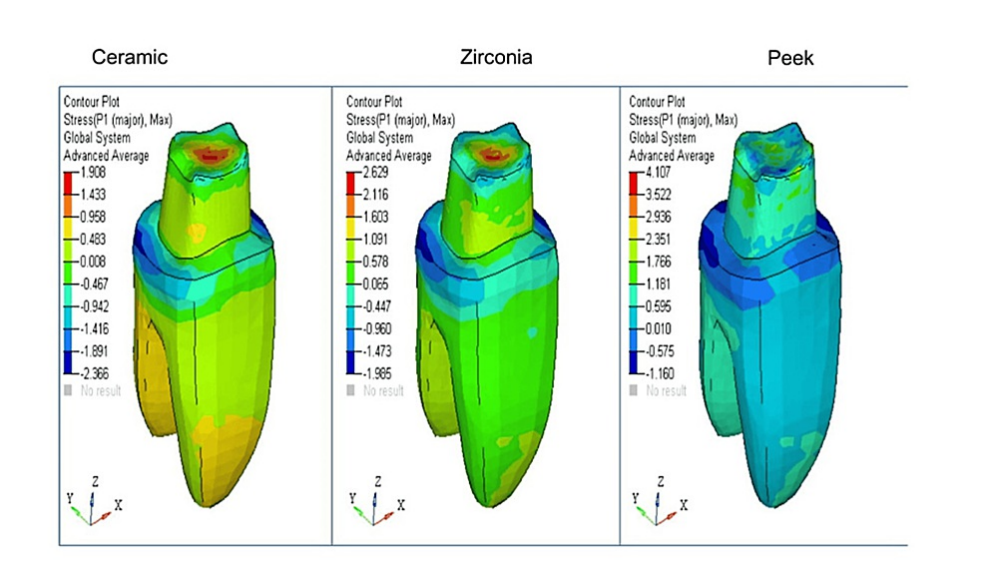
Radial shoulder marginal maximum principal stress contours (MPa)

**Figure 3:**
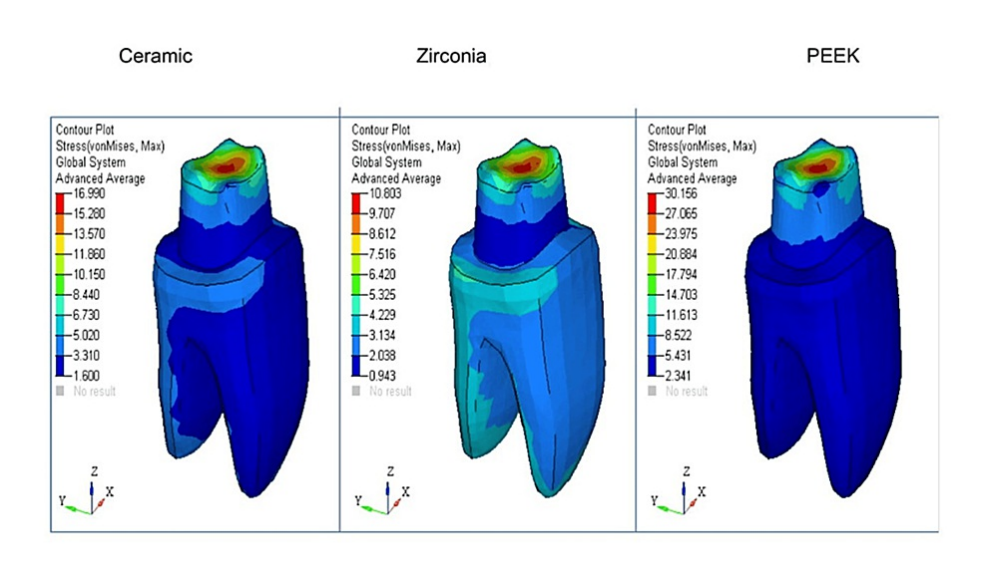
Shoulder margin von Mises stress contours (MPa)

**Figure 4:**
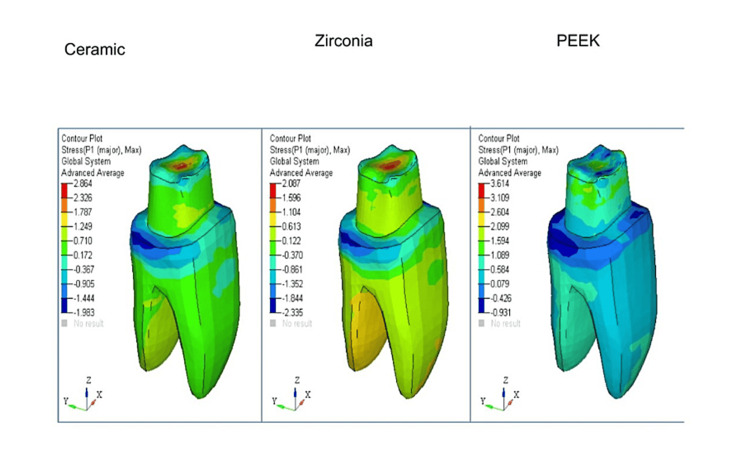
Shoulder margin maximum principal stress contours (MPa)

For easier visual and quantitative comparison of the distribution of stress at the borders of crowns made of various materials, values on a map scale were standardized. Contour plots depicting stress distribution with respect to shoulder and radial shoulder margins for ceramic, zirconia, and PEEK have been represented, respectively (Figure 1, Figure 2, Figure 3, and Figure 4).

In all the models with ceramic, zirconia, and PEEK crowns [4] with shoulder and radial shoulder margins, the occlusal forces of 300N were applied, and the stress distribution analysis was done on 3D FEA models.

von Mises stress comparison was done to help determine if the said material would yield or fracture under load, and tensile stress in FEA was expressed as the maximum principal stress (Pmax).

The maximum and minimum von Mises stress comparisons on crowns with radial shoulder margins are tabulated in Table 2.

**Table 2:** The maximum and minimum von Mises stress comparison on crowns with radial shoulder margins

Part	Ceramic (MPa)	Zirconia (MPa)	PEEK (MPa)
Crown	126.36	125.58	143.41
Dentine	16.99	10.81	30.16
Cementum	6.17	6.21	6.14

The maximum and minimum von Mises stress comparison on crowns with shoulder margins are tabulated in Table 3.

**Table 3:** The maximum and minimum von Mises stress comparison on crowns with shoulder margins

Part	Ceramic (MPa)	Zirconia (MPa)	PEEK (MPa)
Crown	122.71	122.83	133.07
Dentine	10.55	16.94	30.95
Cementum	6.2	6.15	6.15

The maximum and minimum maximum principal stress comparisons on crowns with radial shoulder margins are tabulated in Table 4.

**Table 4:** The maximum and minimum maximum principal stress comparison on crowns with radial shoulder margins

Part	Ceramic (MPa)	Zirconia (MPa)	PEEK (MPa)
Crown	148.86	140.59	123.38
Dentine	1.91	2.63	4.11
Cementum	2.73	2.76	2.82

The maximum and minimum maximum principal stress comparison on crowns with shoulder margins are tabulated in Table 5.

**Table 5:** The maximum and minimum maximum principal stress comparison on crowns with shoulder margins

Part	Ceramic (MPa)	Zirconia (MPa)	PEEK (MPa)
Crown	136.69	145.24	119.46
Dentine	2.86	2.08	3.61
Cementum	2.76	2.74	2.82

It was found that the maximum principal stress was least at the margins of the tooth restored with PEEK crowns, irrespective of the finish line.

## Discussion

The factors that play an important role in the long-term success of crowns are the materials of the crowns, the margins or finish lines of the prepared tooth, and the marginal integrity of said tooth [6]. With increasing demand for aesthetics, crowns without metallic cores or substructures are widely used in clinical practice, with studies concluding that crowns with favorable finish lines provide adequate stress distribution, resulting in maximum and improved durability [7].

This study investigated and compared the influence of two different finish lines on the stress distribution of prepared teeth when crowns of three different materials were used.

All-ceramic crowns are cosmetic dental restorations that are the most natural-looking tooth replacements, are biocompatible, and have superior aesthetics when compared to metal crowns [8]. Zirconia, a dental ceramic crown material, has superior properties of strength and durability when compared to all-ceramic crowns. A randomized controlled trial conducted in 2017 [9] concluded that the crown of this material fared just as well as metal-based crowns, with studies confirming that the material has relatively less cytotoxic effects [10].

With tensile strength similar to that of enamel and dentin, research has suggested that PEEK can be used for the fabrication of dental crowns. The material has the best biocompatibility, making it an ideal material of choice for any restorative purpose. Although aesthetically superior to metals, the material is not as transparent as hybrid ceramic [11].

The border of the preparation where the prepared tooth structure meets the unprepared surface of the tooth is the finish line. A study conducted by Isgrò et al. [12] on the marginal adaptation of fixed prosthodontics concluded that the perfect margin is the most important technical factor for the long-term success of any restoration. Shoulder finish line design is one in which the gingival floor meets the external axial surface at an approximated right angle and is the finish line of choice for all-ceramic crowns and facial margins of metal-ceramic crowns [13]. The wide ledge of the finish line provides resistance to occlusal forces and also provides the required space for healthy restoration contours and maximum aesthetics. Shoulder margins provide the material bulk but are a less conservative preparation, which causes increased stress concentration at 90° internal angles, conducive to coronal fracture [3]. Tekin et al. [14] confirmed in their study that the shoulder margin provides a good seat but a wider marginal seal. Radial shoulder finish lines are characterized by a 120° margin, which is a modification of the shoulder finish line, providing adequate support for the restoration [3]. Ozer et al. [1] conducted a study on the influence of crown margin design on stress distribution on maxillary canines, concluding that the endurance limit with respect to maximum principal stress for both shoulder and radial shoulder margins was similar. Halawani and Al-Harbi [15] concluded in their study that the load-bearing capacity of PEEK crowns was better, making it an alternative crown material for fixed dental prostheses. Within the good aspects of the study, a few shortcomings can be seen, such as intraoral conditions were not simulated as FEA studies are mechanical in nature. The occlusal force applied was not as realistic as it would be in a clinical scenario. The responses obtained have to be validated with further clinical trials.

## Conclusions

In the current study, it is evident that among the crown materials used, maximum principal stress was found in PEEK crowns, irrespective of finish lines, in comparison to ceramic and zirconia crowns. The von Mises stress values were highest for zirconia crowns with shoulder margins and relatively less for PEEK crowns with the same margins as tabulated in Table 2. Hence, PEEK crowns with shoulder or radial shoulder margin are ideal for mandibular first molar rehabilitation with respect to stress distribution and marginal integrity.
